# Weed Density Extraction Based on Few-Shot Learning Through UAV Remote Sensing RGB and Multispectral Images in Ecological Irrigation Area

**DOI:** 10.3389/fpls.2021.735230

**Published:** 2022-03-24

**Authors:** Shubo Wang, Yu Han, Jian Chen, Xiongkui He, Zichao Zhang, Xuzan Liu, Kai Zhang

**Affiliations:** ^1^College of Engineering, China Agricultural University, Beijing, China; ^2^State Key Laboratory of Hydroscience and Engineering, Tsinghua University, Beijing, China; ^3^College of Water Resources and Civil Engineering, China Agricultural University, Beijing, China; ^4^Centre for Chemicals Application Technology, College of Science, China Agricultural University, Beijing, China; ^5^Key Laboratory of Urban Land Resources Monitoring and Simulation, Ministry of Natural Resources, Shenzhen, China

**Keywords:** UAV remote sensing, multispectral, convolutional neural network, bionic optimization, bat algorithm, model-agnostic meta-learning, weeds density

## Abstract

With the development of ecological irrigation area, a higher level of detection and control categories for weeds are currently required. In this article, an improved transfer neural network based on bionic optimization to detect weed density and crop growth is proposed, which used the pre-trained AlexNet network for transfer learning. Because the learning rate of the new addition layer is difficult to tune to the best, the weight and bias learning rate of the newly added fully connected layer is set with particle swarm optimization (PSO) and bat algorithm (BA) to find the optimal combination on the small data set. Data are transported to the convolutional neural network (CNN) by collecting red-green-blue (RGB) and 5-band multispectral images of 3 kinds of weeds and 3 kinds of crops as data sets, through cutting, rotating, and other operations. Finally, 6 kinds of classifications are implemented. At the same time, a self-constructed CNN based on model-agnostic meta-learning (MAML) is proposed in order to realize the learning of neural networks with small sample and high efficiency, and its accuracy is verified in the test set. The neural networks optimized by two bionic optimization algorithms are compared with the self-constructed CNN based on MAML and histogram of oriented gradient + support vector machine (HOG + SVM). The experimental results show that the combination of learning rate through BA is the best, and its accuracy can reach 99.39% for RGB images, 99.53% for multispectral images, and 96.02% for a 6-shot small sample. The purpose of the classification proposed in this article is to calculate the growth of various plants (including weeds and crops) in the farmland. And various plant densities can be accurately calculated through the plant density calculation formula and algorithm proposed in this article, which provides a basis for the application of variable herbicides by experimenting in different farmlands. Finally, an excellent cycle of ecological irrigation district can be promoted.

## Introduction

Ecological irrigation area, an important base for the development of modern agriculture and an important support for regional economic development (Zhang et al., 2011), is also a support for local ecological environment protection (Garnett et al., 2013). An excellent cycle of ecological irrigation district can achieve sustainable use of resources without significant changes to the surrounding environment for a long time while providing advanced productivity capabilities (Fan et al., 2020). To achieve an excellent cycle of irrigation area, one of the conditions is to establish an effective barrier to remove agricultural pollution that is caused by the massive use of agrochemicals (Petit et al., 2015). Extensive spraying of herbicides can control weeds; it not only pollutes the environment and increases the cost of agriculture but also poses a threat to food safety (Chagnon et al., 2015; Delcour et al., 2015). According to the statistics from the Food and Agriculture Organization of the United Nations, there are more than 8,000 varieties of weeds in the world, among which more than 250 varieties can harm crops. Therefore, it is very important to achieve precise weeds prevention and control. Many scholars have conducted researches on weeds extraction. Most of the weed classification methods used at this stage are based on the ground carrier, and complex image processing algorithms are used for feature extraction and classifying, making the method with poor adaptability and robustness. However, convolutional neural networks (CNN) can avoid these problems. The early model of CNN is called the neurocognitive machine, a bio-physical model inspired by the neural mechanism of the visual system. CNN has made a rapid development since its appearance in the field of deep learning showing excellent performance in the field of image recognition (Yanai et al., 2016; Qayyum et al., 2017; Zhi et al., 2018), target location, and detection (Li et al., 2015; Shi et al., 2016; Wu et al., 2017). At present, CNN has been studied by many scholars and applied in many fields. (Zhao et al., 2018) proposed a 15-level CNN called “Fire_Net” that can be implemented effectively in wild-fire detection and classification. (Li et al., 2017) proposed the well-known AlexNet CNN architecture that was utilized in combination with a sliding window object proposal technique for palm tree detection and counting. A CNN method is proposed for weeds classification based on low-altitude plant images captured by the unmanned aerial vehicle (UAV).

Network training can be achieved through a small sample with pre-trained AlexNet based on transfer learning. However, it is difficult to grasp the learning rate parameter setting of the new layer. If the learning rate is set properly, the transfer layer can fine-tune the parameters during the training process, and the newly added layer will quickly adjust the parameters. The network can achieve fast convergence and ultimately find the global optimum. So, it is very important to set the learning rate of the new layer. Bionic optimization is used to optimize the learning rate of the new layer. Bionic intelligent optimization solves complex problems by simulating the functions and behaviors of biological systems in nature. It can be seen as the process of selecting a solution that meets the target requirements as reasonably as possible from many limited or infinite decisions while overcoming the problem in complex solutions that traditional optimization methods cannot solve. At present, the bionic optimization algorithms are divided into evolutionary algorithm (Wu and Wu, 2017), swarm intelligence algorithm (Tian et al., 2016), simulated annealing algorithm (Kong et al., 2015), tabu search algorithm (Fu et al., 2018), and neural network algorithm. Among them, the evolutionary algorithm is divided into genetic algorithm (Jayasinghe et al., 2015), differential evolution algorithm (Zhang et al., 2018), and immune algorithm (Aragón et al., 2015). And the swarm intelligence algorithm is divided into ant colony algorithm (Ren and Sun, 2016) and particle swarm optimization (Kamalova et al., 2017). The swarm intelligence algorithm is a kind of calculation method inspired by the life of the biological group. Because of the excellent cooperation and competition mechanism of the swarm intelligence algorithm, a method based on the bat algorithm (BA) is proposed to optimize the learning rate factor of the transfer neural network. With the accuracy rate as the index of evaluating candidate solutions, the optimal combination of learning rate factors is found through multiple optimization iterations. An improved time factor method is proposed, aiming at the shortcomings of the initial BA, such as a small search range in the early stage and insufficient mining ability in the later stage. Having greatly improved the performance of the transfer neural network, it can accurately extract weeds from the farmland and calculate the density of weeds and crops with red-green-blue (RGB) images. Multispectral images of the same period in this article are collected for comparative experiments.

However, for the new samples, the trained deep CNN often demonstrates performance degradation and must be trained again, which provides poor adaptability. In the training of neural networks, a large number of labeled samples are needed. How to reduce the number of training samples and achieve high accuracy? Meta-learning (ML) can solve the above problems. As a new type of learning strategy, ML can fully rely on past knowledge to guide the learning of new tasks. It is an excellent simulation of human learning methods and an important research direction in the field of neural network classification. As a fast learning method, ML can make neural network adjust itself according to the new task with the previous knowledge, which is very suitable for image classification. Therefore, more and more researchers have carried out research on ML. (Santoro et al., 2016) mainly used the past learning experience to realize the ML and added the additional memory to store the last round of input and tags as input at the same time; consequently, the backpropagation and the previous round of tags and inputs could establish a connection and guide the current round of learning. (Andrychowicz et al., 2016) used the previous task to learn to predict the gradient. In this way, the gradient descent of the neural network would be fast and accurate, achieving the purpose of rapid learning of ML. (Vinyals et al., 2016) used the attention mechanism to perform single-sample learning. Different tasks were weighted with the similarity between the new task and the previous task to obtain the final learning model. In (Sung et al., 2017), the author constructed a model to predict the loss function with the previous tasks and achieved faster learning speed by better loss function. During training, a core value network was first constructed. After encoding previous tasks and combining the current task information, the data were input to the core value network to predict the loss function. Then, the optimization was used to obtain the action operations in enhanced learning or the predicted output in supervised learning. (Finn et al., 2017) proposed a model-agnostic meta-learning (MAML) algorithm, which was independent of the specific model and could be used to solve the problem of small sample learning. In new task solving, only a few training data can get better performance. The core idea of this algorithm was to start multiple tasks at the same time and then to get the synthesis gradient direction of different tasks to update the meta-model. When dealing with a new task, ML training was used to adjust the parameters of the meta-model so that the model could perform well after one or two steps of gradient descent training fine-tuning. In this article, an improved gradient meta-model of inner and outer loop synthesis is proposed with the MAML method. By setting different numbers of external circulation samples and training the meta-model, high-accuracy results have been achieved.

**FIGURE 1:**
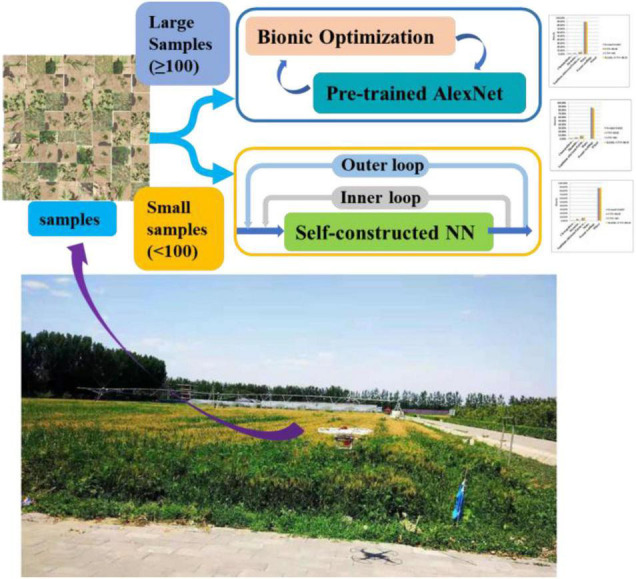
Flowchart of algorithm selection based on different sample sizes.

In general, as shown in Figure 1, two neural network training methods are proposed in this article. One is that when there are a large number of samples, transfer learning and bionic optimization are used to obtain high enough accuracy. The other is that when the sample size is very small, MAML can be used to obtain the suboptimal precision output. The innovations of this article are summarized as follows:

(1) A BA based on improved time factor is proposed to optimize the learning rate factor of the transfer CNN and to realize the plant classification of the bionic optimization neural network.
(2) An improved inner and outer loop synthesis gradient meta-model based on MAML is proposed, aiming at the problem of large number of samples, slow learning speed, and poor generalization of deep CNN training, while finally realizing high precision classification prediction with small sample.

The rest of this article is organized as follows: the “Bionic convolutional neural network” section introduces BA based on the improved time factor and describes the training process of the bionic transfer neural network. The “MAML in CNN” section proposes an improved inner and outer gradient synthesis meta-model based on MAML. The “Data” section makes a series of preprocessing on RGB and 5-band multispectral images of the low-altitude plant to prepare for the input of CNN and ML. The “Experimental results and discussions” section implements the training of bionic optimization neural networks and meta-model networks with different sample sizes and puts forward a method for calculating the density of weeds and experimental results in different farmlands. The “Conclusion” section summarizes the full article.

## Bionic Convolutional Neural Network

**FIGURE 2:**
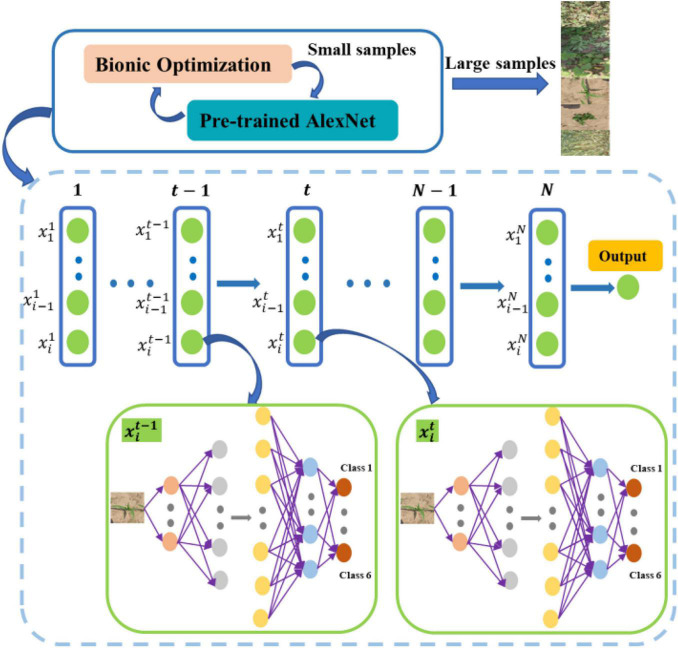
The overall flow of the bionic optimization.

The CNN is used to classify collected plants in this article. Learning rate is an important parameter in supervised learning, which determines whether the loss function can converge to a global minimum and when it converges to a minimum. Transfer CNN is used to achieve 6 classifications by adding new layers. The last 3 layers of AlexNet are modified, and a new fully connected layer, a Softmax layer, and a Classification layer are set up, respectively. Set small learning rates for the transfer layer and fine-tune the network during the training process. The new fully connected layer has the parameters of (4,096 × 6 + 6), and large learning rates need to be set to make the network converge quickly. Setting appropriate learning rates for the new layers is very important for the training time and result of the transfer neural network. If the learning rates are set too small, the convergence process will become very slow and may fall into local optimum. If learning rates are set too large, the gradient will oscillate back and forth around the optimal value and will not converge. The “learning rate factors” for the weight and bias of the fully connected layer are set to 1 by default in MATLAB 2018(a). BA (Yang, 2010) and PSO (Kamalova et al., 2017) are used to optimize the learning rates of the weight and bias of the new layers to obtain the optimal solution. The optimization strategy is to obtain the optimal combination of the learning rate factor parameters by training optimization on the small sample training set and then apply it to the large sample training set to train the neural network. The accuracy is used to evaluate the candidate solution, and the optimal learning rate factor combination is searched through multiple optimization iterations. A more specific description of the newly added learning rate factor is that some solutions are generated during each iteration. The generated learning factor is used to train the neural network whose output accuracy is an evaluation function to select a better solution. As shown in Figure 2, a new solution set is generated through biomimetic iteration, and the neural network is continuously trained and evaluated to finally obtain the optimal learning rate factor after continuous iteration.

### Particle Swarm Optimization

As proposed by Kennedy and Eberhart, 1995 (Kamalova et al., 2017), the idea of the PSO algorithm was derived from the simulation of the foraging process of birds.

Assuming that there are *m* particles in a *N*-dimensional target search space, the position of the *i* first particle can be expressed as *X*_*i*_ = (*x*_*i*1_, *x*_*i*2_, …, *x*_*iN*_), the velocity of the particle is *V*_*i*_ = (*v*_*i*1_, *v*_*i*2_, …, *v*_*iN*_), the individual extreme value is *P*_*i*_ = (*P*_*i*1_, *P*_*i*2_, …, *P*_*iN*_)^*T*^, and the global extreme value is *P*_*g*_ = (*P*_*g*1_, *P*_*g*2_, …, *P*_*gN*_)^*T*^, where *i* = 1, 2, …, *m*. And the update formula for particle velocity and position is:


(1) $$V_{i}^{t+1}=\omega V_{i}^{t}+c_{1}r_{1}\left(P_{i}-X_{i}^{t}\right)+c_{2}r_{2}\left(P_{g}-X_{i}^{t}\right)$$

(2) $$X_{i}^{t+1}=X_{i}^{t}+V_{i}^{t+1}$$

(3) $$\omega =\omega_{\min}+\frac{\left(\omega_{\max}-\omega_{\min}\right)\times \left(\max\text{iter}-t\right)}{\max\text{iter}}$$

where *t* is the current number of iterations; $V_{i}^{t}$ is the velocity of the *i*th particle at the *t*th iteration; $X_{i}^{t}$ is the position of the *i*th particle at the iteration of *t*; *c*_1_ and *c*_2_ are the learning factor; *r*_1_ and *r*_2_ are the random numbers taken between 0 and 1; and ω is the inertia weight. When ω > 1.2, particles can be developed in a larger search space to improve the accuracy of the algorithm. When ω < 0.8, the particles will quickly move closer to the global optimal solution for detailed search in the local range. So, this article uses a linearly decreasing method to ω. ω_*max*_ is the maximum inertia factor, while ω_*min*_ is the minimum inertia factor. And *max* iter is the maximum number of iterations.

**FIGURE 3:**
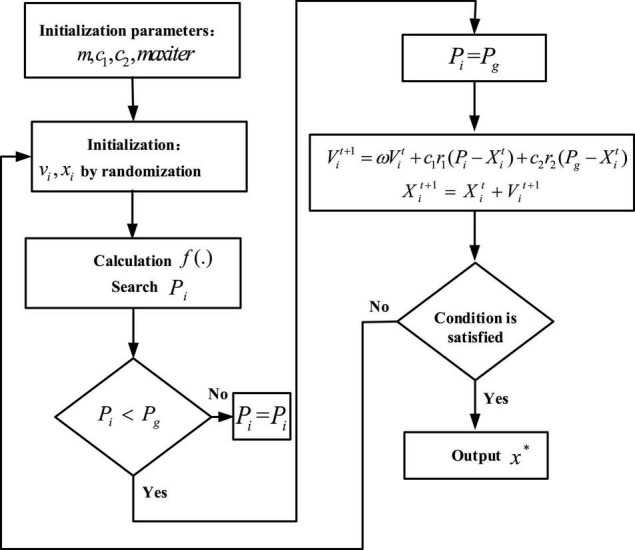
Optimal solution process for PSO.

Figure 3 shows the optimal solution process for the PSO algorithm, and the parameter settings of the algorithm are shown in Table 1.

**TABLE 1:** Parameter settings in PSO.

Subject	*maxiter*	*m*	*c*_1_	*c*_2_	ω_*max*_	ω_*min*_	*V*_*max*_	*V*_*min*_
Parameter	20	30	2	2	1.2	0.8	2	−2

### Bat Algorithm

**FIGURE 4:**
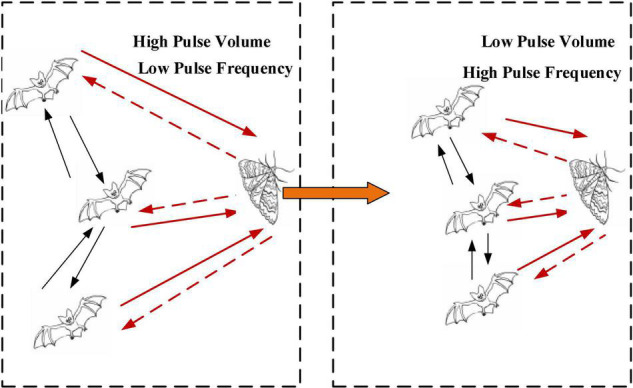
An iterative process for bats looking for food.

Bat algorithm, proposed by X.S. Yang of Cambridge University in 2010 (Yang, 2010), is a new swarm intelligence optimization algorithm, which simulates the behavior of bat echolocation for food exploration. BA mainly determines its calculation and optimization ability by 2 parameters, namely, pulse frequency and pulse volume. In Figure 4, the iterative process for bats looking for food is illustrated.

A time factor improvement method is proposed for the shortcomings of the initial BA with a small search range in the early stage and insufficient mining ability in the later stage. The overall search ability of the algorithm is improved by adding time factor perturbation to the position update equation instead of the implied time factor with constant 1. The time factor disturbance formula (6) and the improved position update formula (7) are as follows:

(1) The pulse frequency of a bat individual while exploring a target.

(4) $$f_{i}=f_{\min}+\left(f_{\max}+f_{\min}\right)\times \text{rand}$$

where *f*_*i*_ is the pulse frequency of the first *i* individual to explore the target, *f*_*min*_ and *f*_*max*_ are the upper and lower limits of the pulse frequency, and rand is a random number between 0 and 1.

(2) The velocity of individual bats in searching for targets.

(5) $$v_{i}^{t}=v_{i}^{t-1}+\left(x_{i}^{t-1}-x^{{}^{*}}\right)\times f_{i}$$

where $v_{i}^{t}$ and $v_{i}^{t-1}$ are the flight velocities of the first *i* individual at time *t* and time *t* − 1, respectively. $x_{i}^{t-1}$ is the position of the bat individual *i* at time *t* − 1.x^*^ is the current optimal position.

(3) Update of the positions of bats.

(6) $$\beta =1+\sin\left(\frac{\pi }{2}-\frac{\pi t}{2t_{\max}}\right)$$

(7) $$x_{i}^{t}=x_{i}^{t-1}+\beta v_{i}^{t}$$

where *t* is the current number of iterations and *t*_*max*_ is the maximum number of iterations.

(4) The update of the pulse frequency and volume of the bat individual in search for prey.

(8) $$r_{i}^{t+1}=r_{i}^{0}\left(1-e^{-\gamma t}\right)$$

(9) $$A_{i}^{t+1}=\alpha A_{i}^{t}$$

where $r_{i}^{0}$ is the maximum pulse frequency, γ is an increase parameter of the pulse frequency and a constant > 0, $A_{i}^{t}$ is the pulse volume of the individual *i* at time *t*, and α is the pulse volume reduction parameter, which is a constant from 0 to 1.

**FIGURE 5:**
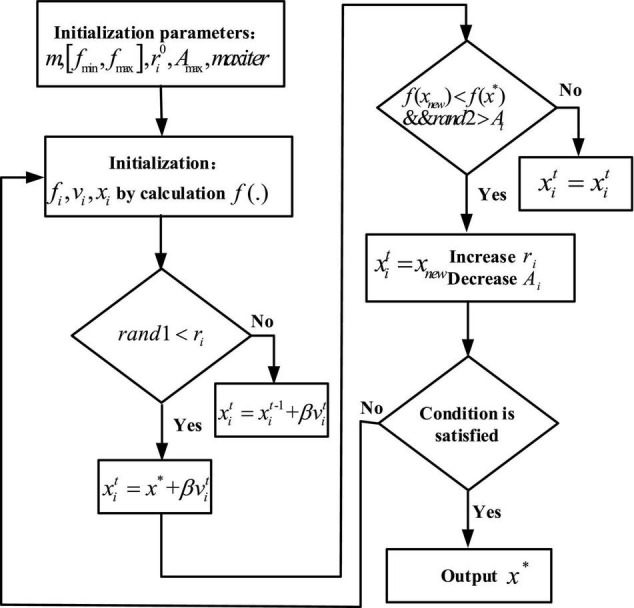
Optimal solution process for BA.

Figure 5 shows the process of the algorithm searching for the optimal solution. In the solution process, it is iteratively closer to the global optimal solution. The learning rates of the weights and bias of the new fully connected layers are set separately, so there are two learning rate parameters whose settings in the algorithm are shown in Table 2.

**TABLE 2:** Parameter settings in BA.

Subject	maxiter	*m*	*f*_*min*_	*f*_*max*_	$r_{i}^{0}$	*A*_*max*_
Parameter	20	10	−1	1	0.85	0.1

### Optimization Result

The classification experiments are run on the MATLAB2018(a) with Deep Learning Toolbox. The hardware environment of the platform is a workstation with double Intel (R) Xeon (R) cpuE5-2620 v4 dual-core CPU, double Nvidia GeForce GTX 1080 Ti, and 64GB memory.

**FIGURE 6:**
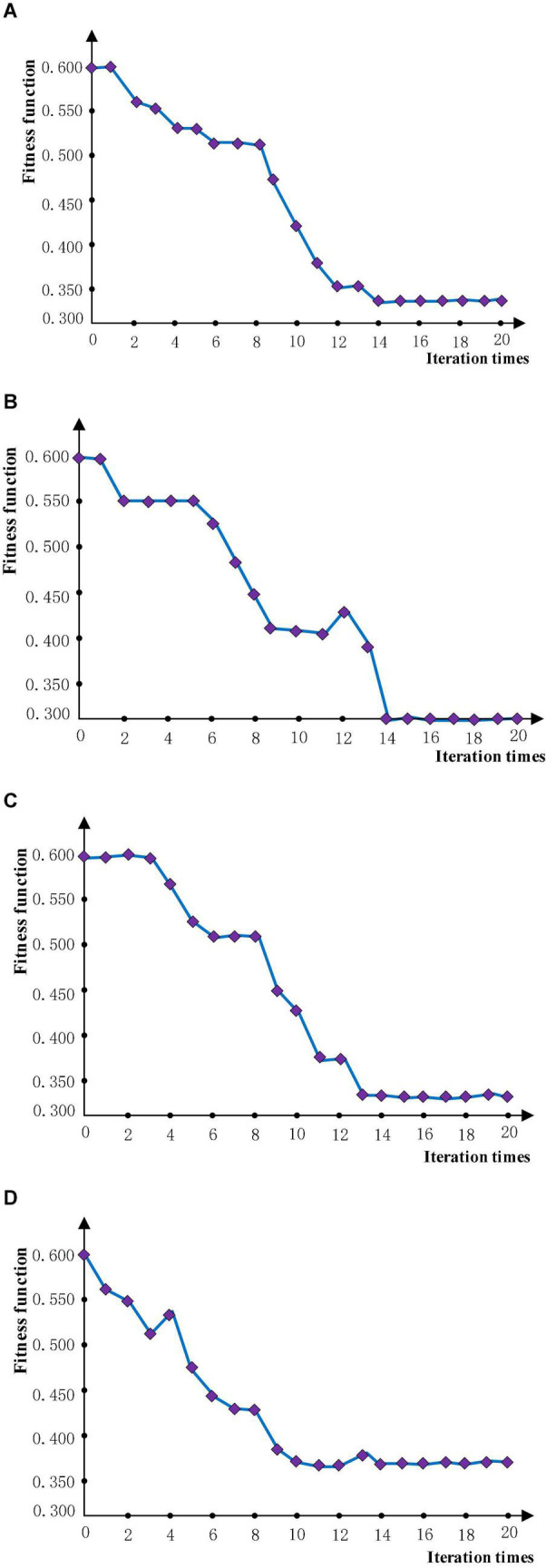
The iterative graph. **(A)** Experiment 1 of PSO. **(B)** Experiment 2 of PSO. **(C)** Experiment 1 of BA. **(D)** Experiment 1 of BA.

The strategy of optimization is to optimize the small sample training set first and then extend the learning rate obtained by optimization to the training of large sample neural network. The accuracy of the neural network trained by the learning rate optimized each time is used as the fitness function after the two optimization methods are performed with 20 iterations and repeated twice. In the training process, 50 sets and 6 different samples are selected for the training set, and 10 sets and 6 types of samples are used in the test set. The number of iterations provided by the neural network is 10 times. The optimization iteration process is shown in Figure 6, where it can be seen that the two optimization methods start to converge after iteration to 15 times, and the fitness function is no longer greatly reduced. Because the concise learning rate parameters can reduce the amount of calculation in the training process, rounding to retain integers is used. The results are averaged as the final optimization results after 2 optimization experiments, and the final optimization results are shown in Table 3.

**TABLE 3:** Results of two optimizations.

Subject	BA		PSO	
	Weight Learn rate factor	Bias Learn rate factor	Weight Learn rate factor	Bias Learn rate factor
First	10.2354	7.6387	6.1143	9.7588
Second	6.3334	9.4124	2.2756	14.0331
Average	8	8	4	12

## MAML Algorithm in CNN

### Model-Agnostic Meta-Learning

The main idea of MAML (Finn et al., 2017) is to find a better initial parameter under which the model can be applied to new tasks with fewer times of iterative training. When a neural network is used to perform classification tasks, the network is trained by starting with initializing random weights and training the network through minimizing losses. To achieve convergence, multiple gradient steps are used to find the optimal weights that are found by learning similarly distributed tasks in MAML. Therefore, for a new task, it is not necessary to start with randomly initialized weights but with optimal weights, thus requiring fewer gradient steps to reach convergence, and the training will not require more data.

**FIGURE 7:**
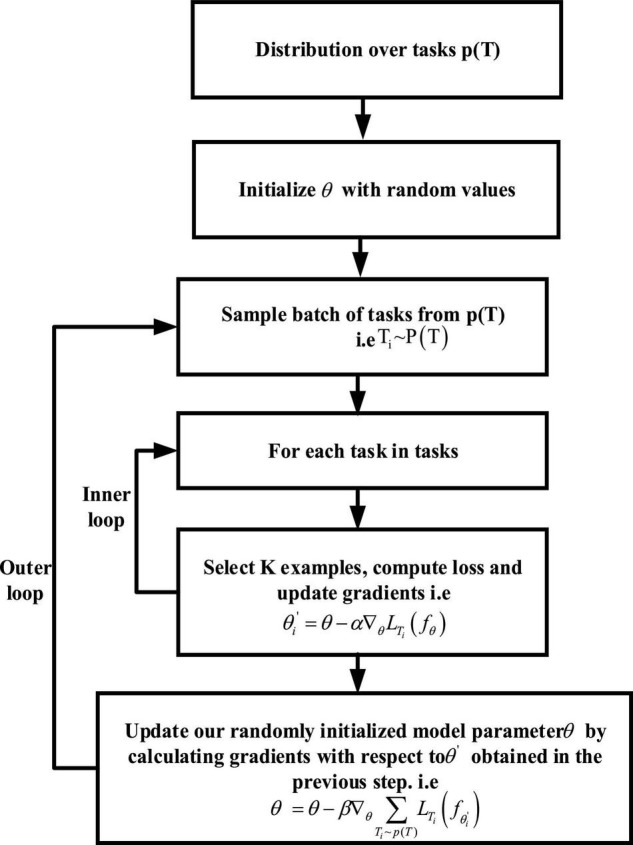
The training process of MAML.

The training process of MAML is as follows: suppose having a parameterized model *f*_θ_ by θ and a distributed *P*(*T*) on the task. First, parameter θ is initialized by some random values. Next, some tasks *T*_*i*_ are extracted from the above tasks, that is *T*_*i*_∼*P*(*T*). Suppose that six tasks T = {*T*_1_, T_2_, *T*_3_, T_4_, *T*_5_, T_6_} are sampled. Then, *k* data points are sampled and used to train the model for each task *T*_*i*_. The optimal parameter to minimize the loss is found by calculation of the loss and use of gradient descent to minimize the loss:


(10) $$\theta_{\text{i}}^{\prime }=\theta -\alpha \nabla_{\theta }L_{T_{i}}\left(f_{\theta }\right)$$

where $\theta_{i}^{\prime }$ is the optimal parameter of the task *T*_*i*_, θ is the initial parameter, α is the super parameter, and ∇_θ_*L*_*T*_*i*__ (*f*_θ_) is the gradient of the task *T*_*i*_. Therefore, after the previous gradient update, the optimal parameters for all 6 tasks will be obtained, and we sampled: $\theta^{\prime }=\left\{\theta_{1}^{{}^{\prime }},\theta_{2}^{{}^{\prime }},\theta_{3}^{{}^{\prime }},\theta_{4}^{{}^{\prime }},\theta_{5}^{{}^{\prime }},\theta_{6}^{{}^{\prime }}\right\}$. Before sampling the next batch of tasks, a meta-update or meta-optimization is performed. That is, in the previous step, the optimal parameter θ is found by training each task *T*. Now the gradient of each task *T* relative to these optimal parameters θ is calculated and the random initialization parameter θ through training is updated. In this way, the random initialization parameter θ is moved to a relatively optimal position to obtain a better initial parameter, and many gradient steps will not be required in the training of next batch of tasks. The whole process can be expressed as follows:


(11) $$\theta =\theta -\beta \nabla_{\theta }\sum_{T_{i}∼p\left(T\right)}L_{T_{i}}\left(f_{\theta_{i}^{\prime }}\right)$$

where θ is the initial parameter, β is the hyperparameter, and $\nabla_{\theta }\sum_{T_{i}∼p\left(T\right)}L_{T_{i}}\left(f_{\theta_{i}^{\prime }}\right)$ is the gradient of each new task *T*_*i*_ to the parameter $\theta_{i}^{\prime }$. Through the parameter updating equation, it can be seen that the model parameter θ can be updated only by taking the average gradient of each new task *T*_*i*_ with the optimal parameter. The whole algorithm flow of MAML can be divided into inner loop and outer loop. In the inner loop, the optimal initial parameter $\theta_{i}^{\prime }$ for different tasks *T*_*i*_ is found through multiple tasks of the same distribution. In the outer loop, the random initial parameter θ is updated on the basis of $\theta_{i}^{\prime }$ obtained, the whole training process of which is given in Figure 7.

### MAML in CNN

This article applies MAML to self-constructed CNN with the finding that the optimal initial parameters can fasten the training speed and improve the generalization of the model. This article performs 6 classifications using cross-entropy loss, as the loss function used is:


(12) $$L_{T_{i}}\left(f_{\theta }\right)=\sum_{x_{j},y_{j}∼T_{i}}y_{i}\log f_{\theta }\left(x_{j}\right)+\left(1-y_{j}\right)\log\left(1-f_{\theta }\left(x_{j}\right)\right)$$

**FIGURE 8:**
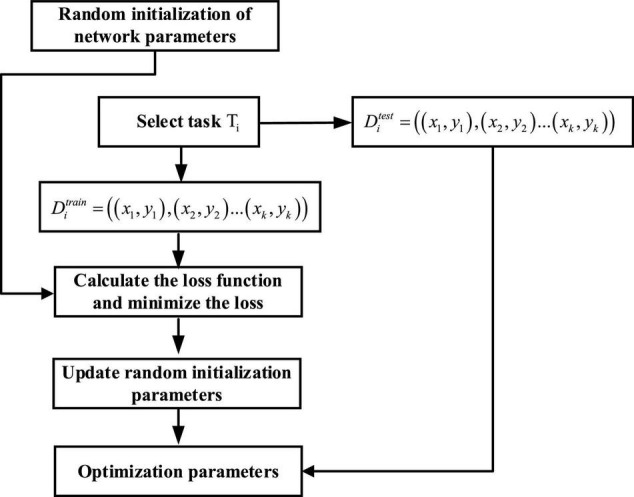
Flowchart of MAML fusion CNN.

The CNN constructed in this article consists of 9 layers, including 4 layers of convolution, 4 layers of pooling, and 1 layer of fully connected layers. The steps for updating MAML in CNN are shown in Figure 8.

In the inner loop, for each task *T*_*i*_ taken from *T*, *k*data points are sampled. The CNN algorithm is used to train*D*_train_, calculate the loss, and then minimize the loss through gradient descent to obtain the optimal parameter $\theta_{i}^{\prime }$, that is, $\theta_{\text{i}}^{\prime }=\theta -\alpha \nabla_{\theta }L_{T_{i}}\left(f_{\theta }\right)$. The same calculation is performed on other tasks, thus obtaining three parameters $\theta_{1}^{\prime }$, $\theta_{2}^{\prime }$, and $\theta_{3}^{\prime }$.

In the outer loop, based on the parameters obtained in the previous step, the meta-test set *D*_test_ uses the following functions to minimize the loss, thus obtaining an optimal initialization parameter:


(13) $$\theta =\theta -\beta \nabla_{\theta }\sum_{T_{i}∼p\left(T\right)}L_{T_{i}}\left(f_{\theta_{\text{i}}^{{}^{\prime }}}\right)$$

## Data

### Image Acquisition

**FIGURE 9:**
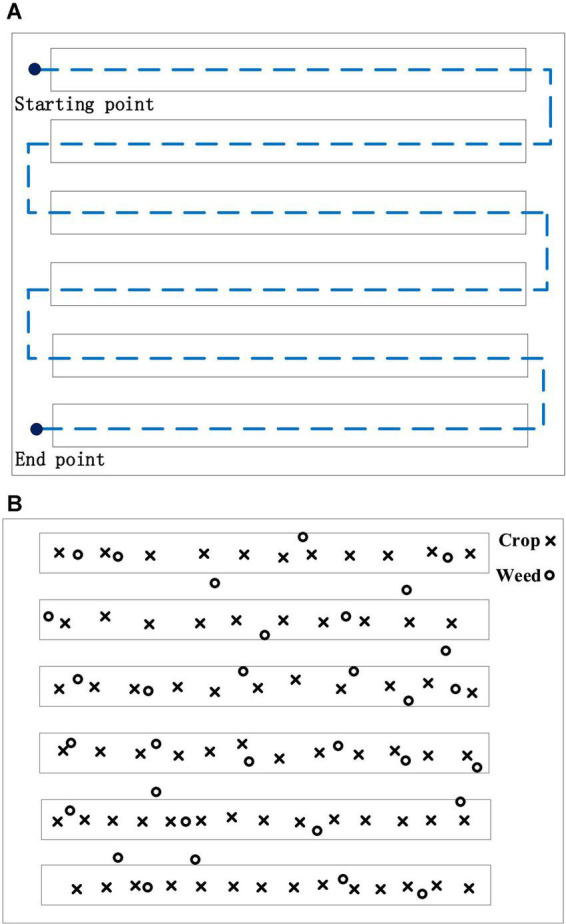
**(A)** Route rules. **(B)** Ground condition.

**FIGURE 10:**
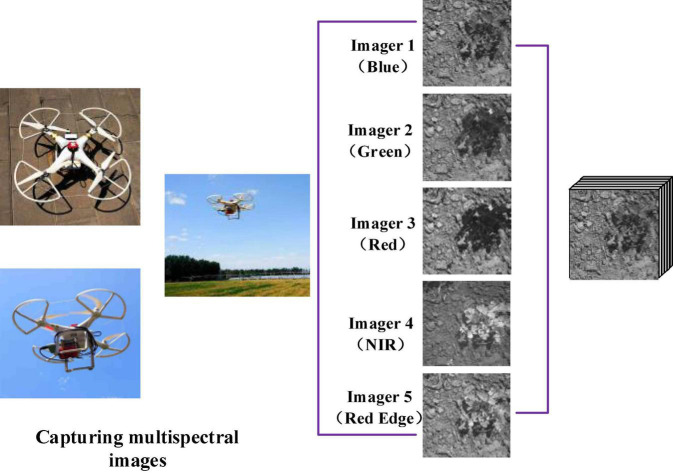
Multispectral image acquisition.

**FIGURE 11:**
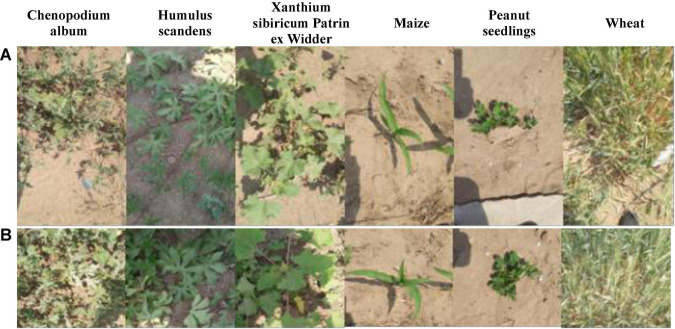
**(A)** Low-altitude captured image. **(B)** Cropped image.

To acquire RGB images, this article adopts the DJI M100 equipped with a ZENMUSE 100 camera (video pixel resolution: 1,920 × 1,080), and to acquire multispectral images, the DJI Phantom 3 Pro is equipped with a RedEdge multispectral camera (Manufacturer: MicaSense, https://micasense.com/atlas). For the spring crops in Zhuozhou farm, Beijing, China (115.84 °E, 39.47 °N), the UAV is set to a height of 2 m for data acquisition. The rule for taking aerial route points and ground conditions is shown in Figure 9., and the flight speed is 1 m/s. The RedEdge installation and collection of multispectral is shown in Figure 10. Although the models of the UAVs used to obtain RGB and multispectral images are different, flight conditions such as flight rules and altitudes are completely consistent. RedEdge obtains 5-band images, and the name and center wavelength of each band are shown in Table 4. The *Chenopodium album*, *Humulus scandens*, maize, peanut seedlings, wheat, *Xanthium sibiricum* Patrin ex Widder, 3 kinds of weeds, and 3 crops are collected, which are used for the classification of crop and weed by CNN. All plant images taken at low altitude as shown in Figure 11A are cut by the square base for subsequent processing.

**TABLE 4:** RedEdge parameters.

Band number	Band	Center wavelength (nm)	Bandwidth FWHM (nm)
1	Blue	475	20
2	Green	560	20
3	Red	668	10
4	Near IR	840	40
5	RedEdge	717	10

### Image Processing

In the process of image acquisition, the UAV is operated to fly in wheat, maize, and peanut fields for about 30 min to obtain ground crop and weed videos. The captured video is intercepted every 0.5 s as a sample set. A total of 3,266 sample sets were obtained. After careful screening by botanists, a sample set is formed as shown in Table 5. Because the AlexNet CNN input image size is 227 × 227, the resolution is also rescaling as 227 × 227. For further expanding data set, the processed image should be rotated by 90°, 180°, and 270°, respectively.

**TABLE 5:** Collection of sample set and label.

Subject	*Chenopodium album*	*Humulus scandens*	*Xanthium sibiricum* Patrin ex Widder	Maize	Peanut seedlings	Wheat	Total
Label	100000	010000	001000	000100	000010	000001	
Train set	370	252	227	490	490	458	2287
Test set	158	108	97	210	210	196	979

**FIGURE 12:**
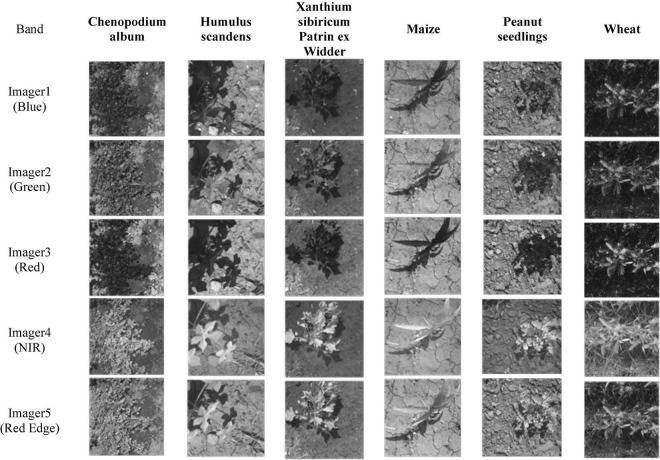
Each band of multispectral image.

Similarly, the acquired multispectral images are cropped, with the cropping rule the same as the RGB images. The 5-band spectra after cropping are shown in Figure 12. Note that for multispectral image samples, one sample contains five different spectrograms, similar to the three-channel color image of RGB. The 5-band multispectral images are also rotated simultaneously for sample expansion. The number of samples must be consistent when multispectral image experiments and RGB image experiments are compared, and the train sets, test sets, and labels are shown in Table 5.

## Experimental Results and Discussion

### Classification Experiments and Results

The learning rate combinations and default values obtained by the two bionic optimizations are iteratively trained on the double GPUs mentioned above to obtain an optimal neural network, and the neural network obtained by optimization has a better training effect than the neural network with the default learning parameters. Even if the number of iterations reaches 144, the accuracy rate is still below 80%. In comparison with the default values, the two kinds of bionic optimization neural network training results are better. Through comparison of the 144 iteration graphs of BA and the PSO, the performance of the iterative process of the BA is found to be better than that of the PSO. In summary, the BA has the best effect in 144 iterations of the neural network, that is, the selection of the parameters of the neural network is optimal at this time. In contrast experiment, the optimal transfer neural network (BA, 144 iterations) is trained to a multispectral sample set where the accuracy of the final display is listed in Table 6.

**TABLE 6:** Accuracy and time of every method.

Method		CNN + RGB						CNN + MS	MAML + CNN + RGB (Finn et al., 2017)		HOG + SVM (Radman et al., 2017)
		PSO (Kamalova et al., 2017)		BA (Yang, 2010)		Default (MathWorks, 2021)		BA	2-Shot	6-Shot	
Parameters	Learning rate factor	(8,8)		(4,12)		(1,1)		(4,12)			
	Iteration	72	144	72	144	72	144	144	9,600	9,600	
Time (s)		68	116	68	116	68	116	196	11	25	400
Accuracy		93.46%	98.47%	95.20%	99.39%	38.51%	71.20%	99.53%	68.62%	96.02%	69.46%
											

The method of changing the number of samples is used to carry out the comparative experiment in the classification experiment of self-constructed CNN based on MAML (Finn et al., 2017). One sample of the inner loop and one sample of the outer loop are used for the 2-shot experiment. Five samples of the inner loop and one sample of the outer loop are used for the 6-shot experiment. After iteration of 9,600 times for experiments, the trained neural network is put into the test set in Table 6 to calculate the prediction accuracy. The experimental results show that the best effect of the 6-shot is 96.02% and that of the 2-shot is 68.62%. In comparison with CNN + RGB and CNN + MS, the accuracy is reduced by about 4%. However, in terms of training time, the advantages of MAML + CNN are obvious with only 25 s in training time in the 6-shot experiment.

Histogram of oriented gradient (HOG) feature combined with support vector machine (SVM) classifier has been widely used in image recognition, especially in pedestrian detection. This article uses HOG + SVM to train and test on the data set of Table 6 for further comparison with the HOG + SVM experiment (Radman et al., 2017). HOG feature, a feature descriptor used for object detection in computer vision and image processing, consists of calculating and counting the gradient direction histogram of the local area of the image. The cell size is set to 6 × 6 pixels, and the block size is set to 2 × 2 with 50% overlap. The basic model of SVM is used to find the best separating hyperplane in the feature space to maximize the interval between positive and negative samples in the training set.

### Estimation Results of the Density of Weeds

The premise of an excellent cycle of ecological irrigation area is to accurately grasp the growth and density of plants in the farmland. The growth of plants can be understood by calculating the density of plants. In the process of classification of weeds, the results can be used to estimate the density of weeds. To achieve the calculation of the density of weeds, this article uses the ratio of the number of weeds to the total low-altitude image. The formula is given as follows:


(14) $$\rho =\frac{\sum_{j=1}^{m}X_{i}^{j}}{\sum_{i=1}^{n}\sum_{j=1}^{m}X_{i}^{j}}$$

where *j* represents the number of samples of a type of plant, *i* represents the total types of plants, and ρ represents the density of weeds. The density of weeds in ecological irrigation areas can be conveniently calculated at a low cost through this formula.

The accuracy of the density measurement algorithm is verified in three different farmlands (i.e., maize, wheat, and peanut). The continuous RGB and 5-band multispectral images collected in three farmlands (i.e., Farmland 1, Farmland 2, and Farmland 3) are taken as experimental objects; the images are manually classified, and the results of which are as shown in Ground truth 1, Ground truth 2, and Ground truth 3 in Table 7. The continuous RGB and multispectral images collected in three blocks are, respectively, input into the optimal neural network and MAML + CNN, and the density value is obtained with the above density calculation algorithm; the density calculation results are shown in Table 7.

**TABLE 7:** The density calculation results of different farmlands.

Subject	*Chenopodium album*	*Humulus scandens*	*Xanthium sibiricum* Patrin ex Widder	Maize	Peanut seedlings	Wheat
Farmland 1	5	5	10	162	0	0
Ground truth 1	2.75%	2.75%	5.49%	89.01%		
CNN+RGB	2.20%	2.75%	4.95%	90.11%		
CNN+MS	2.20%	2.75%	6.04%	89.01%		
MAML+CNN+RGB	2.20%	2.20%	7.69%	87.91%		
Farmland 2	3	5	15	0	151	0
Ground truth 2	1.72%	2.87%	8.62%		86.78%	
CNN+RGB	1.15%	4.02%	7.47%		87.36%	
CNN+MS	2.30%	3.45%	8.62%		85.63%	
MAML+CNN+RGB	2.87%	4.02%	9.20%		83.91%	
Farmland 3	2	7	12	0	0	140
Ground truth 3	1.24%	4.35%	7.45%			86.96%
Experiment 3	1.24%	4.35%	6.83%			87.58%
CNN+MS	1.24%	3.73%	8.07%			86.96%
MAML+CNN+RGB	1.24%	2.48%	8.07%			88.20%

### Discussion

This research highlights the use of end-to-end neural networks to extract plant information and calculate density. Two neural network training methods are adopted: one is the transfer neural network based on bionic optimization and the other is the neural network based on MAML. Among them, the bionic optimization neural network training is to obtain high enough accuracy when there are many samples. When the sample size is very small, MAML (Finn et al., 2017) can be used to obtain the suboptimal precision output. Through comparative experiments, it can be seen from Table 6 that the optimized neural network by BA (Yang, 2010) is best. The accuracy rate is 99.39% for RGB images, and the accuracy rate is 99.53% for multispectral images. PSO (Kamalova et al., 2017) is the second. MAML + CNN is the third with the accuracy reaching 96.02%. HOG+SVM (Radman et al., 2017) has the worst effect with an accuracy rate of 69.46%. This also shows that HOG is not capable of extracting static object features in comparison with CNN. In the small sample training, the accuracy of the self-constructed CNN based on MAML reaches 96.02% when the sample size of each class is 6. Although the accuracy of large samples is 4% higher than the 6-shot, the difference between the two is negligible in the calculation of plant density. And the training time of a large sample is 5 times that of a 6-shot, greatly consuming GPU resources, which is very disadvantageous for offline computing.

**FIGURE 13:**
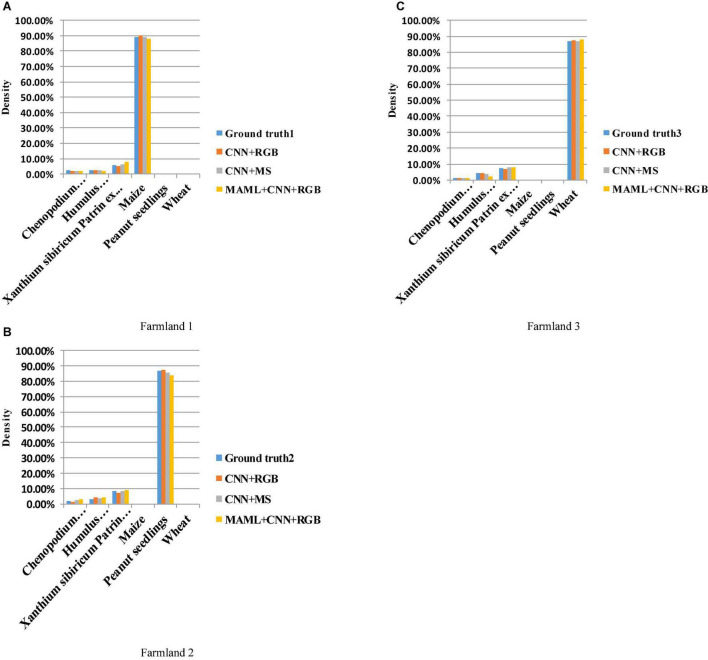
**(A)** Density histogram of Farmland 1.**(B)** Density histogram of Farmland 2. **(C)** Density histogram of Farmland 3.

After multiple sets of simulation comparisons, the transfer CNN with learning parameters of (4, 12) and iterations of 144 times is finally selected in the bionic optimization neural network experiment. The 6-shot (inner loop 5, outer loop 1) is selected in the experiments of self-constructed CNN based on MAML. Three farmlands are selected for the final practice to verify the reliability of the algorithm and used low-cost RGB and high-cost multispectral images as experimental data. It can be seen from Table 7 that the three methods have a very small difference from the Ground truth. The density calculation results presented in this article can provide a basis for the application of variable spraying of herbicides. As shown in Figure 13, a histogram is used to show the density of the three farmlands for a more visual display of the results.

## Conclusion

The low-altitude RGB and multispectral images captured, respectively, by UAVs were used, and the classification of weeds was implemented with bionic optimization neural network and self-constructed CNN based on MAML. PSO and BA were used to optimize the selection for the learning rate setting of the weights and bias of the new layer. The combination of the proposed algorithm of the density of weeds can effectively obtain the density of various weeds, which can be established to provide a reference for the application of variable spraying of herbicides in the later stage. In turn, it can reduce the use of herbicides and the production costs of agricultural products, improve food safety, and ultimately achieve an excellent cycle of ecological irrigation area. There are corresponding limitations: only 3 crops and 3 weeds are collected, and as a result, only the classification and density calculation of these 6 plants can be performed. Plant data of more species and more growth periods can be collected to facilitate a further extension of the method, which will be the next research direction of this method.

## Data Availability Statement

The raw data supporting the conclusions of this article will be made available by the authors, without undue reservation.

## Author Contributions

SW, YH, ZZ, XL, and KZ contributed to the conceptualization, methodology, software, validation, formal analysis, investigation, resources, data curation, writing—original draft, writing—review and editing, and visualization. JC contributed to the conceptualization, methodology, software, validation, formal analysis, investigation, resources, data curation, writing—original draft, writing—review and editing, visualization supervision, funding acquisition, and project administration. All authors contributed to the article and approved the submitted version.

## Conflict of Interest

The authors declare that the research was conducted in the absence of any commercial or financial relationships that could be construed as a potential conflict of interest.

## Publisher’s Note

All claims expressed in this article are solely those of the authors and do not necessarily represent those of their affiliated organizations, or those of the publisher, the editors and the reviewers. Any product that may be evaluated in this article, or claim that may be made by its manufacturer, is not guaranteed or endorsed by the publisher.

## Abbreviations

CNN: Convolutional neural network
ML: Meta-learning
MAML: Model-agnostic meta-learning
RGB: Red-green-blue
BA: Bat algorithm
PSO: Particle swarm optimization
MS: Multispectral
UAV: Unmanned aerial vehicle
HOG: Histogram of oriented gradient
SVM: Support vector machine.

## References

[B1] Andrychowicz M. Denil M. Gomez S. Hoffman M. W. Pfau D. Schaul T. (2016). “Learning to learn by gradient descent by gradient descent,” in *Proceedings of the 30th International Conference on Neural Information Processing Systems* (Barcelona), 3981–3989.

[B2] Aragón V. S. Esquivel S. C. Coello C. A. (2015). An immune algorithm with power redistribution for solving economic dispatch problems. *Inf. Sci.* 295 609–632. 10.1016/j.ins.2014.10.026

[B3] Chagnon M. Kreutzweiser D. Mitchell E. A. D. Morrissey C. A. Noome D. A. Van der Sluijs J. P. (2015). Risks of large-scale use of systemic insecticides to ecosystem functioning and services. *Environ. Sci. Pollut. Res.* 22 119–134. 10.1007/s11356-014-3277-x 25035052 PMC4284381

[B4] Delcour I. Spanoghe P. Uyttendaele M. (2015). Literature review: impact of climate change on pesticide use. *Food Res. Int.* 68 7–15. 10.1016/j.foodres.2014.09.030

[B5] Fan X. Zhang W. Chen W. Chen B. (2020). Land–water–energy nexus in agricultural management for greenhouse gas mitigation. *Appl. Energ.* 265:114796.

[B6] Finn C. Abbeel P. Levine S. (2017). “Model-agnostic meta-learning for fast adaptation of deep networks,” in *Proceedings of the 34th International Conference on Machine Learning-Volume 70. JMLR. Org* (Sydney, NSW), 1126–1135. 10.1016/j.neunet.2021.09.029

[B7] Fu Q. Zhou K. Qi H. Jiang F. (2018). A modified tabu search algorithm to solve vehicle routing problem. *Comput. Soc. Republic China* 29 197–209.

[B8] Garnett T. Appleby M. C. Balmford A. Bateman I. J. Benton T. G. Bloomer P. (2013). Sustainable intensification in agriculture: premises and policies. *Science* 341 33–34. 10.1126/science.1234485 23828927

[B9] Jayasinghe J. M. Jeevani W. Anguera J. Uduwawala D. N. Andújar A. (2015). Nonuniform overlapping method in designing microstrip patch antennas using genetic algorithm optimization. *Int. J. Antennas Propagation* 2015:805820.

[B10] Kamalova D. I. Galimullin D. Z. Sibgatullin M. E. Salakhov M. K. (2017). An evolutionary particle swarm optimization algorithm for mathematical processing of experimental spectra. *Optics Spectrosc.* 122 687–691. 10.1134/s0030400x17050101

[B11] Kennedy J. Eberhart R. (1995). “Particle swarm optimization,” in *Proceedings of the IEEE International Conference on Neural Networks*, Vol. 4 Perth, WA, 1942–1948.

[B12] Kong X. H. Zheng Y. L. Qin G. Q. Li R. H. (2015). An improved simulated annealing algorithm for dynamic grid scheduling. *Int. J. Simulation Syst. Sci. Technol.* 16 13.1–13.5. 10.1186/s13638-018-1061-1 31258613 PMC6566211

[B13] Li S. D. Tang H. He S. Shu Y. Mao T. Li J. (2015). Unsupervised detection of earthquake-triggered roof-holes from UAV images using joint color and shape features. *IEEE Geosci. Remote Sens. Lett.* 12 1823–1827. 10.1109/lgrs.2015.2429894

[B14] Li W. J. Fu H. H. Yu L. Cracknell A. (2017). Deep Learning based oil palm tree detection and counting for high-resolution remote sensing images. *Remote Sens.* 9:22. 10.3390/rs9010022

[B15] MathWorks (2021). *Pretrained AlexNet Network Model for Image Classification.* Natick, MA: MathWorks.

[B16] Petit S. Munier-Jolain N. Bretagnolle V. Bockstaller C. Gaba S. Cordeau S. (2015). Ecological intensification through pesticide reduction: weed control, weed biodiversity and sustainability in arable farming. *Environ. Manag.* 56 1078–1090. 10.1007/s00267-015-0554-5 26071767

[B17] Qayyum A. Malik A. S. Saad N. M. Iqbal M. Faris Abdullah M. Rasheed W. (2017). Scene classification for aerial images based on CNN using sparse coding technique. *Int. J. Remote Sens.* 38 2662–2685. 10.1080/01431161.2017.1296206

[B18] Radman A. Zainal N. Suandi S. A. (2017). Automated segmentation of iris images acquired in an unconstrained environment using HOG-SVM and GrowCut. *Digit. Signal Process.* 64 60–70.

[B19] Ren W. D. Sun W. X. (2016). Application of an improved ant colony algorithm in TSP problem solving. *Chem. Eng. Transac.* 51 373–378.

[B20] Santoro A. Bartunov S. Botvinick M. Wierstra D. Lillicrap T. P. (2016). “Meta-learning with memory-augmented neural networks,” in *Proceedings of the 33rd International Conference on International Conference on Machine Learning - Volume 48* (New York, NY), 1842–1850.

[B21] Shi W. W. Gon Y. H. Wang J. J. Zheng N. N. (2016). “Integrating supervised laplacian objective with CNN for object recognition,” in *Proceedings of the 17th Pacific-Rim Conference on Multimedia* (Xi’an), 64–73. 10.1007/978-3-319-48896-7_7

[B22] Sung F. Zhang L. Xiang T. Hospedales T. Yang Y. (2017). *Learning to Learn: Meta-Critic Networks for Sample Efficient Learning. Arxiv [Preprint].* Available online at: https://arxiv.org/abs/1706.09529 (accessed April 1, 2021).

[B23] Tian D. X. Hu J. J. Sheng Z. G. Wang Y. P. Ma J. M. Wang J. (2016). Swarm intelligence algorithm inspired by route choice behavior. *J. Bionic Eng.* 13 669–678.

[B24] Vinyals O. Blundell C. Lillicrap T. Kavukcuoglu K. Wierstra D. (2016). Matching networks for one shot learning. *Adv. Neural Inf. Process. Syst.* 29 3630–3638.

[B25] Wu H. Bie R. F. Guo J. G. Meng X. Zhang C. Y. (2017). CNN refinement based object recognition through optimized segmentation. *Optik* 150 76–82. 10.1016/j.ijleo.2017.09.071

[B26] Wu X. L. Wu S. M. (2017). An elitist quantum-inspired evolutionary algorithm for the flexible job-shop scheduling problem. *J. Intell. Manuf.* 28 1441–1457.

[B27] Yanai K. Tanno R. Okamoto K. (2016). “Efficient mobile implementation of A CNN-based object recognition system,” in *Proceedings of the 2016 ACM Multimedia Conference* (New York, NY), 362–366.

[B28] Yang X. S. (2010). A new metaheuristic bat-inspired algorithm. *Comput. Knowledge Technol.* 284 65–74.

[B29] Zhang G. H. Xing G. Y. Cao F. (2018). Discrete differential evolution algorithm for distributed blocking flowshop scheduling with makespan criterion. *Eng. Application Artificial Intell.* 76 96–107.

[B30] Zhang Z. Li Y. Zhang W. Xu J. Gu H. Qi Q. (2011). “Discuss on the ecological irrigation district and ecological irrigation district health,” in *Proceedings of 2011 International Symposium on Water Resource and Environmental Protection* (Xi’an), 489–493.

[B31] Zhao Y. Ma J. L. Li X. H. Zhang J. (2018). Saliency detection and deep learning-based wildfire identification in UAV imagery. *Sensors* 18:712. 10.3390/s18030712 29495504 PMC5876738

[B32] Zhi S. F. Liu Y. X. Li X. Guo Y. (2018). Toward real-time 3D object recognition: a lightweight volumetric CNN framework using multitask learning. *Comput. Graph.* 71 199–207.

